# A Neglected Case of Massive Urinary Ascites Secondary to Posterior Urethral Valve: A Developing World’s Scenario 

**Published:** 2012-07-01

**Authors:** Kanchan Kayastha, Bilal Mirza

**Affiliations:** Department of Pediatric Surgery, The Children’s Hospital and the Institute of Child Health, Lahore, Pakistan.

**Dear Sir**

Developing countries are not only lagging behind in health facilities but also literacy of the population. Many uneventfully manageable conditions use to present after complications have been occurred. Negligence of the poor people and their blind faith on the fraudulent quacks and peers add burden to the poor health facilities in the resource poor countries. This could be one of important reasons of higher mortality rate in our hospitals especially in neonates with poor reserves to combat these crises for long. Urinary ascites due to in-utero bladder perforation secondary to posterior urethral valves is a rare entity. This condition is being prevented in developed countries by Fetendo which involves decompression of the urinary bladder by vesico-amniotic shunting or by endoscopic in-utero valve ablation [1]. For instance if bladder perforation has occurred, it can be amenable to drainage of urinary ascites with valve ablation [2]. However, we received a delayed presenting case of in-utero bladder perforation with massive urinary ascites secondary to posterior urethral valve necessitating urgent intervention. 

A 20-day-old male neonate, weighing 2.1kg, presented to our neonatal emergency with the history of massive abdominal distension and failure to pass urine since birth. The baby was a product of consanguineous marriage and born via spontaneous vaginal delivery in home setting. The patient passed stool within few hours of the birth and also tolerated the breast feed. The baby however did not pass urine for that parents urgently took the child to a quake that advised circumcision as a therapy of urinary retention. The circumcision was performed by the barber but patient still did not pass urine. The baby was taken to multiple “peers”, “Hakeem’s” and quacks but patient did not pass urine and his abdominal distension worsened. The patient became reluctant to feeds thence the parent brought the child to the city where a local practitioner referred the case to our tertiary care centre.

At presentation, the baby was dehydrated with temperature 100F, pulse of 140/min, and respiratory rate of 60/min. Abdomen was tense, shiny, and red around umbilicus. Rectal examination reveled stool in it. Patient was resuscitated with NG tube decompressions, intravenous fluids and broad spectrum antibiotics. A resistance was felt while passing feeding tube as urinary catheter. X-ray and ultrasound of abdomen were performed which revealed massive ascites, however; the bladder was thick walled and not distended along with no hydroureteronephrosis. The BUN and creatinine level were in normal ranges. Exploratory laparotomy was performed for rapid deterioration of the patient. On exploration there was a perforation of the intraperitoneal part of urinary bladder with about 500 ml of urine in peritoneal cavity (Fig.1). Rest of the gut and abdominal viscera were normal. Tube vesicostomy was performed as an initial procedure to stabilize the patient. Patient showed uneventful recovery. Posterior urethral valve were fulgurated at three months follow up. The patient has lost to follow up after 6 months of regular follow-up.

**Figure:**
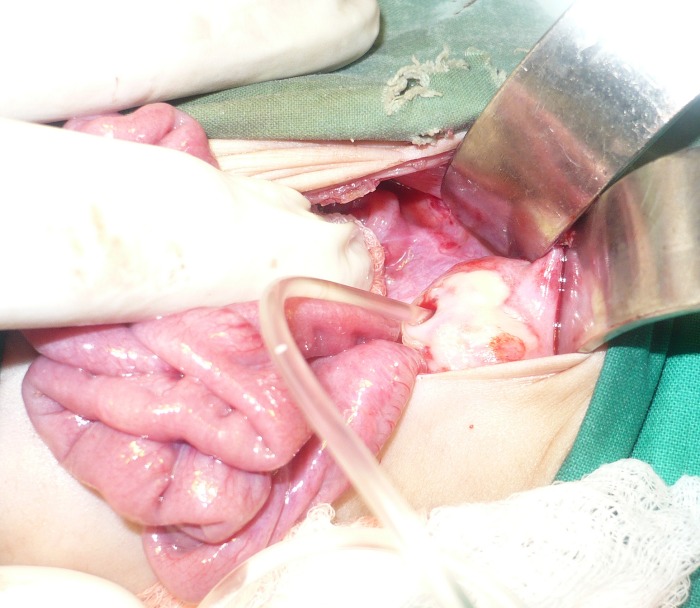
Figure 1: Showing a feeding tube passed to the urinary bladder through the perforation.

In case of fetal urinary ascites mother usually present with oligohydramnios [2]. Due to poor socioeconomic condition, the mother never underwent any antenatal ultrasound scanning and patient born with massive urinary ascites due to bladder perforation. Bladder perforation acted as pop off valve to prevent development of hydroureteronephrosis in our case. This was also the reason of normal blood urea and creatinine in our patient. The prognosis of bladder perforation secondary to posterior urethral valve is good if the patient is managed in time with proper urinary drainage and valve fulguration/ablation [3]. Our patient is an exemplary scenario of ignorance and improper attitude towards acquiring appropriate health services in developing world.

## Footnotes

**Source of Support:** Nil

**Conflict of Interest:** None

